# Quadruplex Real-Time TaqMan^®^ RT-qPCR Assay for Differentiation of Equine Group A and B Rotaviruses and Identification of Group A G3 and G14 Genotypes

**DOI:** 10.3390/v15081626

**Published:** 2023-07-26

**Authors:** Mariano Carossino, Udeni B. R. Balasuriya, Côme J. Thieulent, Maria E. Barrandeguy, Maria Aldana Vissani, Viviana Parreño

**Affiliations:** 1 Louisiana Animal Disease Diagnostic Laboratory, School of Veterinary Medicine, Louisiana State University, Baton Rouge, LA 70803, USA; cthieulent@lsu.edu; 2 Department of Pathobiological Sciences, School of Veterinary Medicine, Louisiana State University, Baton Rouge, LA 70803, USA; 3 Escuela de Veterinaria, Universidad del Salvador, Buenos Aires B1630, Argentina; maria.barrandeguy@usal.edu.ar (M.E.B.); vissani.aldana@inta.gob.ar (M.A.V.); 4 Instituto de Virología, CICVyA, Instituto Nacional de Tecnología Agropecuaria (INTA), Buenos Aires B1686, Argentina; parreno.viviana@inta.gob.ar; 5 Consejo Nacional de Investigaciones Científicas y Técnicas (CONICET), Buenos Aires C1425, Argentina

**Keywords:** rotavirus A, equine rotavirus A, ERVA, rotavirus B, equine rotavirus B, ERVB, one-step quadruplex RT-qPCR, G-typing, G3, G14, foal diarrhea

## Abstract

Equine rotavirus A (ERVA) is the leading cause of diarrhea in foals, with G3P[12] and G14P[12] genotypes being the most prevalent. Recently, equine G3-like RVA was recognized as an emerging infection in children, and a group B equine rotavirus (ERVB) was identified as an emergent cause of foal diarrhea in the US. Thus, there is a need to adapt molecular diagnostic tools for improved detection and surveillance to identify emerging strains, understand their molecular epidemiology, and inform future vaccine development. We developed a quadruplex TaqMan^®^ RT-qPCR assay for differentiation of ERVA and ERVB and simultaneous G-typing of ERVA strains, evaluated its analytical and clinical performance, and compared it to (1) a previously established ERVA triplex RT-qPCR assay and (2) standard RT-PCR assay and Sanger sequencing of PCR products. This quadruplex RT-qPCR assay demonstrated high sensitivity (>90%)/specificity (100%) for every target and high overall agreement (>96%). Comparison between the triplex and quadruplex assays revealed only a slightly higher sensitivity for the ERVA NSP3 target using the triplex format (*p*-value 0.008) while no significant differences were detected for other targets. This quadruplex RT-qPCR assay will significantly enhance rapid surveillance of both ERVA and ERVB circulating and emerging strains with potential for interspecies transmission.

## 1. Introduction

Equine rotavirus A (ERVA) is considered the leading cause of diarrhea in neonatal foals, causing up to 77% of foal diarrhea cases worldwide [1,2,3,4,5,6,7,8,9]. The high fecal viral shedding from infected foals and the high resistance in the environment contribute to their rapid transmission to other susceptible animals and environmental persistence [1,2,3,4,5,6,7]. ERVA infection causes life-threatening diarrhea in young foals and has a high morbidity rate. It is, therefore, considered a major health problem to equine breeding enterprises with significant economic impact [6]. In 2021, a group B equine rotavirus has been recognized as a cause of foal diarrhea (see below, [10]). This emerging virus could potentially serve as an additional, significant cause of diarrhea in foals; however, further epidemiological analysis is required to determine its impact.

Rotaviruses are icosahedral, non-enveloped viruses in the family *Sedoreoviridae* (genus *Rotavirus*) based on the most recent report by the International Committee on Taxonomy of Viruses (ICTV; [11,12,13]). Their double-stranded RNA genome consists of 11 discrete linear RNA segments encoding for six structural (VP1, 2, 3, 4, 6, and 7) and six non-structural viral proteins (NSP1 through 6). Segment 11 has two overlapping open reading frames (ORFs) and its translation yields the non-structural proteins NSP5 and NSP6 [1,13,14]. The icosahedral virion is composed of a triple-layered capsid constituted by an outer capsid consisting of VP7 and VP4, an intermediate layer formed by VP6 and an inner capsid formed by VP2 and the minor structural proteins VP1 (RNA-directed RNA polymerase) and VP3 (capping enzyme) [15,16,17,18]. The VP6 is highly conserved and carries species-specific determinants bearing epitopes that allow antigenic classification into groups/species (A-L), among which group A is the most common cause of diarrhea in humans and animals [19,20,21]. Both VP7 and VP4 are the most variable and immunogenic viral proteins and elicit neutralizing antibodies [22,23,24]. Based on the nucleotide identity of these two outer capsid proteins, group A rotaviruses are further classified into G-types and P-types [25]. Among the seven G-types (G3, G5, G6, G8, G10, G13, and G14) and six P-types (P[1], P[3], P[7], P[11], P[12], and P[18]) recognized in ERVA strains, the G3P[12] and G14P[12] genotypes are by far considered the most prevalent and epidemiologically relevant in the US and globally [1,4,26,27,28,29,30]. In recent years, novel equine-like G3 strains have been identified in children around the world, demonstrating that emerging reassortants derived from rotaviruses affecting animal species can emerge, jump species (zoonosis), and consequently have a public health impact [31,32,33,34]. Hence, surveillance tools for ERVA strains are also important from a public health perspective. During the 2017 foaling season, our team undertook a major surveillance effort in Central Kentucky, determining that ERVA G14 strains are the most prevalent in this region [30].

Disease control in horses relies on routine vaccination of pregnant mares with a partially protective, inactivated vaccine in order to provide protective colostral immunity to newborn foals as well as on strict husbandry/hygienic practices aimed at reducing viral burden in the environment [1,6,35,36,37,38]. The current vaccine used in the US includes an inactivated G3 ERVA type only (G3P[12], strain H-2) dating over two decades [36]. Multiple studies undertaken since have identified antigenic variation among ERVA genotypes, possibly compromising vaccine efficacy [30,39,40,41,42,43], which is also impacted by the temporal and spatial variations in circulating ERVA strains [4,30,44]. Consequently, continued surveillance of ERVA is critical to collect genotypic data, understand its molecular epidemiology, identify novel viral reassortants with emerging potential, allow early detection of interspecies transmission, and assess vaccine performance in the field.

In 2021, an emergent equine group B rotavirus (ERVB) was identified as the etiological agent involved in localized outbreaks of diarrhea in Central Kentucky [10]. Infections associated with RVB have been extensively reported in diarrheic pigs, ruminants, and humans, while occurrence in horses has only been rarely reported [45,46,47,48,49,50,51]. In contrast to RVA, RVB outbreaks in humans have not been linked to direct viral transmission from animals [52]; however, the biology of RVB remains poorly understood compared to RVA and this possibility needs to be further investigated. While the disease caused by RVB resembles that of RVA gastroenteritis, RVB seemingly affects primarily adult humans rather than pediatric populations [53]; factors determining infection of this age category are unknown. RVB has a similar genome composition as RVA, with 10 out of 11 segments predictively encoding proteins with RVA homologs [54,55]. In contrast, the segment encoding the NSP1 of RVB differs significantly and contains two overlapping open reading frames (ORFs) whose encoded protein products have little predicted sequence or structural homology with known proteins [56,57]. A classification for G genotypes of RVB strains from the US similar to that for RVA has been proposed in 2012 [46] and expanded by Shepherd et al. [51], who established a provisional genome-based classification for RVB, proposed 26G, 5P, 13I, 5R, 5C, 5M, 8A, 10N, 6T, 4E, and 7H genotypes (VP7, VP4, VP6, VP1-VP3, and NSP1-NSP5 genes, respectively) of RVB.

Single RT-qPCR assays for ERVB were rapidly developed [10]; however, molecular assays that can simultaneously differentiate ERVA from ERVB are needed to strengthen surveillance efforts and make these assays most cost-effective. Our laboratory has previously developed and validated a one-step multiplex (triplex) TaqMan^®^ RT-qPCR assay that allows both detection as well as genotypification of ERVA in a single reaction by targeting NSP3, G3 VP7, and G14 VP7 gene segments of ERVA [58]. Here, we converted this triplex assay into a quadruplex RT-qPCR assay for the simultaneous differentiation of ERVA and ERVB, and genotyping of G3 and G14 ERVA in feces. This new multiplex RT-qPCR assay has a performance nearly equivalent to the previously developed assay, as well as with conventional ERVA VP7-specific and ERVB VP6-specific RT-PCR and Sanger sequencing.

## 2. Materials and Methods

### 2.1. Cell Lines, Viruses, and Viral RNA

MA-104 cells (ATCC^®^ CRL-2378.1^™^, American Type Culture Collection [ATCC], Manassas, VA, USA) were maintained as previously described [30]. Tissue culture fluid (TCF) derived from MA-104 cells infected with ERVA strain H2 (G3P[12]), ERVA strains RVA/Horse-tc/ARG/E8701-5MCCH/2016/G14P[12], RVA/Horse-tc/ARG/E8701-6MCBI/2016/G14P[12], and RVA/Horse-tc/ARG/E8701-9MCGR/2016/G14P[12]; bovine RVA (BRVA) strain NCDV-Lincoln (G6P[1]), BRVA strain B223 (G10P[11]), porcine RVA (PRVA) strain OSU (G5P[7]), PRVA strain Gottfried (G4P[6]), simian RVA strain SA11 (G3P[2]), human RVA strain Wa (TC-adapted, G1P[8]; ATCC VR-2018), human RVA strain Hu/Australia/1-9-12/77/S (G2P[4], ATCC VR-1546), and RVA reassortant WI79-4 (G6P[8], ATCC VR-2377) were used to assess specificity of the developed RT-qPCR assays as previously described [30]. RNA from the following viruses that cause diarrhea in horses were included for specificity evaluation of the newly developed quadruplex RT-qPCR assay: TCF containing ERVA strains RVA/Horse-tc/GBR/H2/1976/G3P[12], RVA/Horse-tc/ARG/E8701-5MCCH/2016/G14P[12], RVA/Horse-tc/ARG/E8701-6MCBI/2016/G14P[12], and RVA/Horse-tc/ARG/E8701-9MCGR/2016/G14P[12] [30]; TCF containing equine coronavirus strain NC99 [59], and TCF containing equine rhinitis A (NVSL-0600EDV8501, National Veterinary Services Laboratory, Ames, IA, USA) and B (NVSL-0610EDV85010, National Veterinary Services Laboratory) viruses.

### 2.2. Bacterial DNA

DNA from the following bacteria that cause diarrhea in horses were included for specificity evaluation of the newly developed quadruplex RT-qPCR assay: *Escherichia coli*, *Salmonella enterica*, *Rhodococcus equi*, *Neorickettsia risticii*, *Clostridium perfringens*, *Clostridium difficile*, and *Lawsonia intracellularis*. These were kindly provided by Dr. Erdal Erol, University of Kentucky Veterinary Diagnostic Laboratory (Lexington, KY, USA).

### 2.3. Fecal Samples

One-hundred and ninety-three fecal samples from diarrheic foals were included in this study. From these, 128 were either collected from farms in Central Kentucky during the 2017 foaling season (n = 112 [30]) or during an outbreak of diarrhea in 2021 (n = 16), and 65 were derived from outbreaks previously reported in Argentina [30,44]. Fecal suspensions were prepared as previously described and stored at −80 °C [30].

### 2.4. Transmission Electron Microscopy (TEM)

Fecal samples from the 2021 diarrhea outbreak (n = 16) were diluted to 10% in deionized water and separated into two aliquots. The first aliquot was clarified by a 2 min centrifugation at 12,000× *g* and a formvar, carbon-coated, 400-mesh copper grid was floated on a 50 µL drop of the supernatant for 15 min. Excess supernatant was removed from the grid with filter paper and stained with 3% aqueous phosphotungstic acid, pH 7.0, for 1 min. The second aliquot was first centrifuged at 1000× *g* for 15 min and the supernatant was subsequently centrifuged at 10,000× *g* for 30 min and 40,000× *g* for 1 h. The supernatant was discarded, and the pellet was resuspended in purified deionized water and a 50 µL drop of the resuspension was stained as indicated above. Grids were viewed with a JEOL JEM-1011 Transmission Electron Microscope at an accelerating voltage of 100 kV (JEOL USA, Inc., Peabody, MA, USA). Representative viral particles were digitally imaged using an XR80M Wide-Angle Multi-Discipline Mid-Mount CCD Camera from AMT (Advanced Microscopy Techniques, Woburn, MA, USA).

### 2.5. Nucleic Acid Isolation

Nucleic acid isolation was performed and stored as previously described using the taco™ mini nucleic acid extraction system (GeneReach USA, Lexington, MA, USA) [30,60].

### 2.6. RT-PCR Amplification of ERVA VP7 (Segment 9) and ERVB VP6 Genes (Segment 6) and Sanger Sequencing for G-Typing

ERVA VP7-specific (gene segment 9) and ERVB VP6-specific (gene segment 6) standard RT-PCR assay and sequencing were performed as previously described [61] and used as the gold-standard method for ERVA and ERVB detection in fecal specimens [4,62]. A high-fidelity RT-PCR kit (QIAGEN One-Step *Ahead* RT-PCR kit, Qiagen, Hilden, Germany) was used for generating full-length amplicons of ERVA VP7 and partial amplicons of ERVB VP6 for sequencing as previously described [30]. DNA was submitted for Sanger sequencing to GeneLab, Louisiana State University, School of Veterinary Medicine. Both DNA strands of ERVA VP7 or ERVB VP6 amplicons were sequenced using a panel of primers (Table S1). Sequences were analyzed with Geneious R7 (Biomatters Inc., Newark, NJ, USA).

### 2.7. Accession Numbers

The nucleotide sequences derived from the fecal samples and tissue culture fluid corresponding to ERVA strains utilized in this study were previously deposited in GenBank under accession numbers MG970165-MG970197, MH458234-MH458237, KP116019-KP116049, and MF074190-MF074212. ERVB VP6 nucleotide sequences were deposited under accession numbers OP314521-OP314535.

### 2.8. Primer and Probe Design

Previously developed ERVA-specific primers and probes with modified dyes were used in this study (Table 1). ERVB VP6 and NSP5-specific forward and reverse primers and probes were designed as previously described [58]. Primer and probe sequences are shown in Table 1.

### 2.9. Synthesis of ERVA and ERVB In Vitro Transcribed RNA for Analytical Performance Evaluation

For ERVA, a previously synthesized in vitro transcribed (IVT) RNA with a 493 nt insert containing the targeted regions (NSP3, G3 VP7, and G14 VP7) was prepared and used as described [58]. A similar approach was used to develop ERVB IVT RNA containing the target sequences in a 214 bp insert (VP6 [nt position 132–230] and NSP5 [nt position 124–238] from ERVB isolate RVB/Horse-wt/USA/KY1518/2021 (GenBank Accession numbers MZ327693.1 and MZ327698.1, respectively). Cloning, preparation of IVT RNA and determination of the number of ERVA and ERVB IVT RNA molecules per microliter (copies/μL) were performed as we previously described in detail [58]. IVT RNA stock (10^7^ copies/μL) was serially ten-fold diluted (10^7^–10^0^ IVT RNA copies/μL) in nuclease-free water and 40 ng/μL Ambion^®^ Yeast tRNA (ThermoFisher Scientific, Waltham, MA, USA).

### 2.10. ERVA and ERVB-Specific Multiplex TaqMan^®^ Real-Time RT-PCR Assays Targeting G3 VP7, G14 VP7 and NSP3 Genes of ERVA, and VP6 or NSP5 Genes of ERVB

The G3 VP7, G14 VP7, and NSP3-specific assays were multiplexed as previously described [58]. In addition, ERVB VP6- or NSP5-specific primers and probes (Table 1) were included to generate two different quadruplex assays (namely, ERVA/ERVB VP6 or ERVA/ERVB NSP5 quadruplex). The reaction was set up using the QuantiTect™ Multiplex RT-PCR kit (Qiagen) in a 25 μL reaction containing 12.5 μL of 2× QuantiTect™ Multiplex RT-PCR Master Mix with ROX, 0.25 μL QuantiTect™ RT Mix, 200 nM of each TaqMan^®^ fluorogenic probe, 200 nM of each primer, and 5 μL of template RNA previously denatured at 95 °C for 5 min. An ABI 7500 Fast Real-time PCR System (Applied Biosystems^®^, Waltham, MA, USA) was used with the following program: 20 min at 50 °C (reverse transcription step), 15 min at 95 °C (PCR initial activation step), and 40 cycles at 94 °C for 45 s (denaturation) and 60 °C for 75 s (combined annealing/extension) [58].

### 2.11. Statistical Analysis

ERVA or ERVB IVT RNA (10^7^ to 10^0^ IVT RNA copies/μL) were used to generate standard curves. For analytical performance, regression analysis, coefficients of determination (*R*^2^), and PCR amplification efficiencies (%) were calculated as previously described [58]. The limit of detection with 95% confidence (LOD_95%_) was determined by probit analysis (IBM SPSS Statistics, Chicago, IL, USA) with 12 subsequent replicates per dilution near the detection limit (10^4^–10 IVT RNA copies/μL) performed on a separate day. Precision (within-run and between-run imprecision) of the ERVA/ERVB VP6 or ERVA/ERVB NSP5 quadruplex assays was determined as previously described [58] with 12 replicates on the same run (within-run imprecision) or three replicates tested on two different operational days. The coefficient of variation (CV %) was determined for each target (VP6, NSP5, NSP3, G3, and G14). Cycle threshold (Ct) cut-off values were determined as the average Ct + 3 standard deviations of 12 replicates of the endpoint dilution [64]. Clinical performance of the ERVA/ERVB-VP6 quadruplex RT-qPCR assay was evaluated in fecal specimens and compared to the ERVA VP7-specific RT-PCR, ERVB VP6-specific RT-PCR, and G-typing by Sanger sequencing as well as previously recorded results from the ERVA triplex RT-qPCR assay [58]. Contingency tables (2 × 2) were generated to determine the sensitivity, specificity, and agreement (weighted kappa index) of each target within the ERVA/ERVB-VP6 quadruplex RT-qPCR assay. For the agreement analysis, the weighted kappa index was calculated. This index gives different weights to disagreements according to the magnitude of the discrepancy avoiding the weakness of the kappa statistic that takes no account of the degree of disagreement. Values of weighted kappa from 0.41 to 0.60 indicate moderate agreement; values from 0.61 to 0.80 substantial agreement and values from 0.81 to 0.99 almost perfect agreement [65]. Differences in the performance of the previously developed triplex and the newly developed quadruplex RT-qPCR assays were tested using McNemar’s test on JMP16 Pro (JMP, Cary, NC, USA). Statistical significance was set at *p*-value < 0.05.

## 3. Results

### 3.1. Analysis of Fecal Samples Included in this Study by Standard RT-PCR and TEM

A total of 193 fecal samples were included in the study, from which 177 were archived samples used in previous studies [30,58] and 16 were derived from a recent outbreak (2021 foaling season) of diarrhea in foals from Central Kentucky during which ERVB was first identified [10]. Of the 193 samples, 93 samples were confirmed negative for ERVA and ERVB, 85 were positive for ERVA as determined by VP7-specific standard RT-PCR [30,44] and 15 (derived from the 2021 foaling season) were positive for ERVB by VP6-specific standard RT-PCR. From the 85 ERVA-positive samples, 41 and 44 were confirmed as G3 or G14 genotypes by sequencing of the VP7 gene, respectively. The ERVB-positive samples derived from the 2021 foaling season (n = 15) were subjected to TEM. Rotaviral particles were evident in a total of seven samples (1 through 5, 9, and 13 [Figure S1]).

### 3.2. Analytical Performance of ERVA and ERVB-Specific Multiplex TaqMan^®^ RT-qPCR Assays Targeting ERVA NSP3, G3 VP7, G14 VP7 and ERVB VP6 or NSP5

#### 3.2.1. Analytical Sensitivity and Specificity of ERVA/ERVB-VP6-Specific Multiplex RT-qPCR Assay

**Figure 1:**
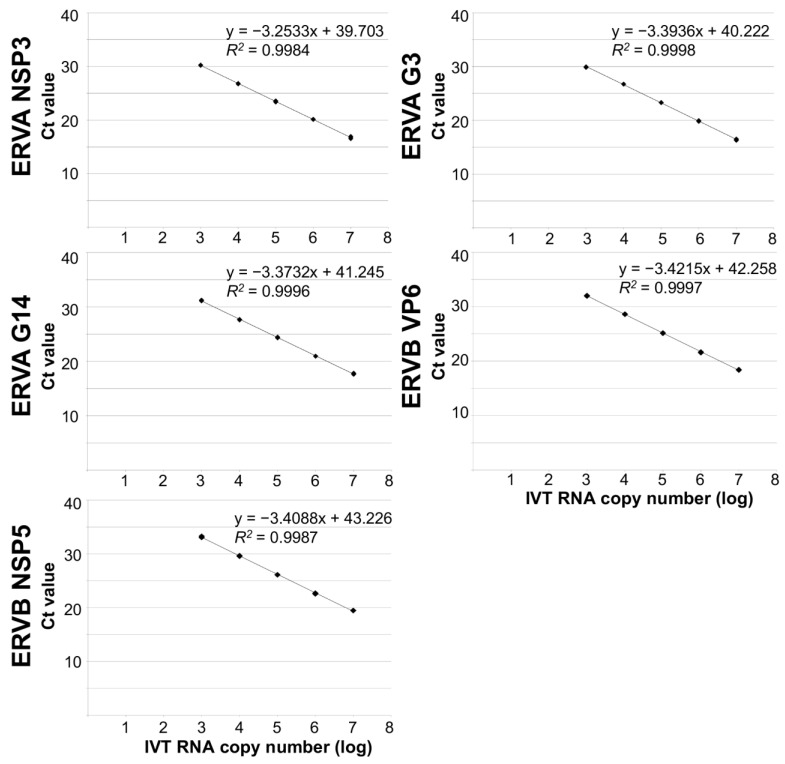
Comparison of the analytical sensitivity of singleplex ERVA (NSP3, G3, and G14) and ERVB (VP6, NSP5) RT-qPCR assays. Data from the ERVA singleplex assays were adapted from our previous publication [58]. Ct, cycle threshold; IVT RNA, in vitro transcribed RNA.

**Figure 2:**
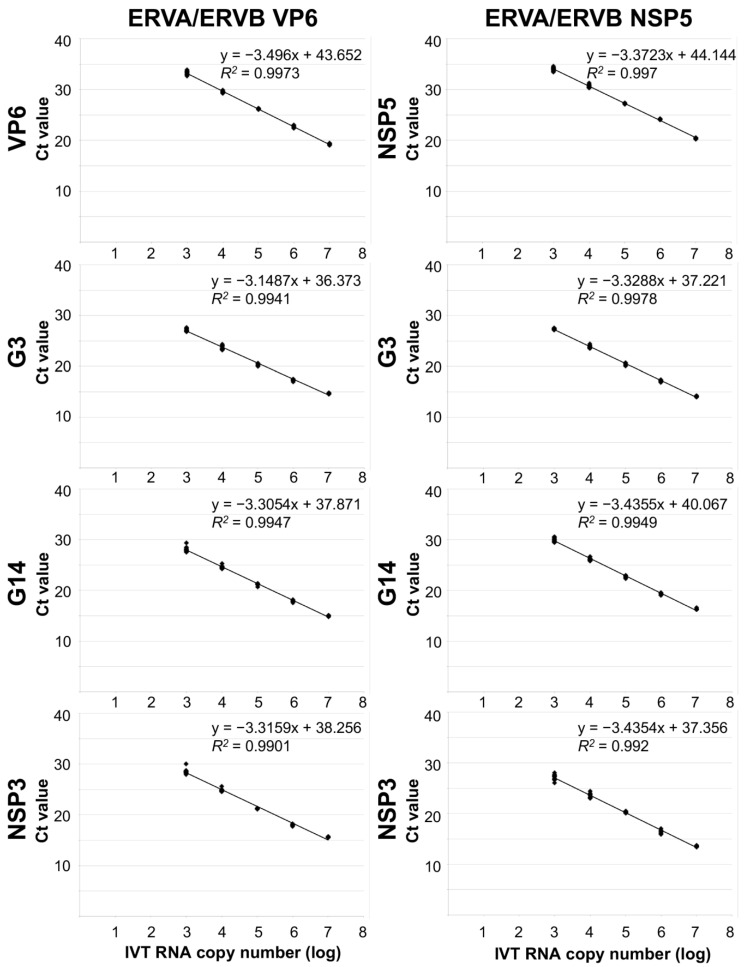
Comparison of the analytical sensitivity of the ERVA/ERVB VP6 and ERVA/ERVB NSP5 quadruplex RT-qPCR assays. Ct, cycle threshold; IVT RNA, in vitro transcribed RNA.

The analytical sensitivity of the ERVA/ERVB-VP6-specific multiplex RT-qPCR assays was determined using a ten-fold serial dilution series (6–12 replicates per dilution) of IVT RNA (10^7^ to 10^0^ IVT RNA copies/μL) containing the target sequences. Standard curves were generated for each of the four targets on the linear range (G3 VP7, G14 VP7, ERVA NSP3, and ERVB VP6). Performance parameters of single and quadruplex assays are summarized in Table 2, Figure 1 and Figure 2. Perfect linearity (*R*^2^ > 0.99, Table 2 and Figure 2) and amplification efficiencies of 108%, 100%, 100% and 93%, respectively, were confirmed. The LOD was determined to be 10^2^ and 10^3^ copies/μL of IVT RNA for the three ERVA targets and ERVB VP6, respectively. Compared to the singleplex ERVB VP6-specific assay, there is a 10-fold difference in the detection rate (Table 2). A panel of rotavirus strains along with other viruses and bacteria associated with diarrhea in horses was used to assess the analytical specificity as described under Materials and Methods. The ERVA/ERVB VP6 quadruplex assay proved to be specific for detection of group A rotaviruses of various animal species and human (via the RVA NSP3 target, serving as a pan-group A rotavirus assay), as well as specific for the respective ERVA genotypes G3 and G14 and ERVB targets and did not amplify other viruses or bacteria associated with diarrhea in horses. The ERVA genotyping targets (G3 and G14 VP7) performed as previously reported, with no cross-reactivity between each other. No cross-reactivity between ERVA and ERVB detection was noted.

#### 3.2.2. Analytical Sensitivity and Specificity of ERVA/ERVB-NSP5-Specific Multiplex RT-qPCR Assay

The analytical sensitivity of the ERVA/ERVB-NSP5-specific multiplex RT-qPCR assay was determined as described above for the ERVA/ERVB VP6 quadruplex assay and results are summarized in Table 2. This assay also demonstrated perfect linearity (*R*^2^ > 0.99, Table 2 and Figure 2) and equivalent LOD, but amplification efficiencies were overall lower across targets compared to the ERVA/ERVB VP6 quadruplex assay (Table 2). Similar to the ERVB VP6-specific singleplex assay, there is a 10-fold difference in the detection rate between the singleplex ERVB NSP5-specific assay and the ERVA/ERVB NSP5 quadruplex assay (Table 2). The assay’s specificity was equal to that of the ERVA/ERVB VP6 quadruplex assay, and no off-target amplification was noted.

#### 3.2.3. Precision Assessment of ERVA/ERVB VP6 and ERVA/ERVB NSP5-Specific Multiplex RT-qPCR Assays

To determine assays’ precision, both within-run and between-run imprecision were determined. In all cases, CV was less than 3%, indicating that both assays have high repeatability (within-run) and reproducibility (between-run) (Table 3).

### 3.3. Clinical Performance of the ERVA/ERVB VP6-Specific Multiplex RT-qPCR Assay Targeting ERVA NSP3, G3 VP7, G14 VP7 and ERVB VP6 Genes

**Figure 3:**
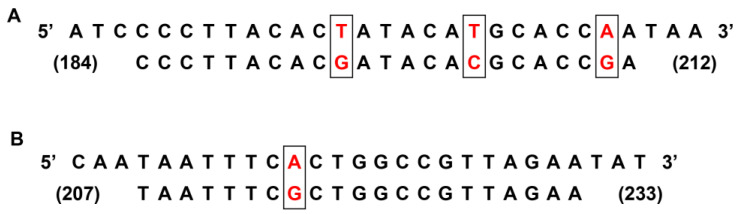
Nucleotide sequence alignment between the RVB/Horse-wt/USA/KY1-6/2021 sequence (**A**,**B**, **top strand**) and the ERVB-VP6-P probe (**A**, **bottom strand**) or the ERVB-VP6-R reverse primer (**B**, **bottom strand**) nucleotide sequences. The probe and primer sequences are based on the reference ERVB VP6 gene sequence (GenBank Accession Number MZ327693.1). Nucleotide differences between the field strain RVB/Horse-wt/USA/KY1-6/2021 and the probe and reverse primer sequences are boxed and in red font. Nucleotide positions are numbered between parentheses. No nucleotide differences were noted between the field strain sequences and the ERVB-VP6-F forward primer (not shown).

Based on the overall higher analytical efficiency across targets of the ERVA/ERVB VP6 quadruplex assay compared to that of the ERVA/ERVB NSP5 quadruplex assay (Table 2), the former was selected for further evaluation of its clinical performance using a total of 193 fecal samples. Overall, the new ERVA/ERVB VP6 quadruplex assay correctly identified most of the fecal samples with only a few exceptions (Table 4a–d) and a high level of agreement compared to RT-PCR (96.4–99.5% and kappa 0.926–0.985). The specificity for all targets in the ERVA/ERVB VP6 quadruplex assay was 100% compared to RT-PCR, with no non-specific amplifications observed in negative samples. The NSP3 (pan-RVA) assay showed a sensitivity of 91.8% when compared to RT-PCR. In the case of the G3 and G14 VP7 targets, the ERVA/ERVB VP6 quadruplex assay was able to correctly genotype 38/41 ERVA G3 samples and 44/45 ERVA G14 samples (Table 4b,c) when compared to RT-PCR and Sanger sequencing, yielding a sensitivity of 92.7% and 97.8%, respectively. Regarding detection of ERVB, the ERVA/ERVB VP6 quadruplex assay was able to correctly detect ERVB in 14/15 samples (sensitivity of 93.3%; Table 4d). Two of the positive ERVB samples (RVB/Horse-wt/USA/KY1-6/2021 and RVB/Horse-wt/USA/KY1-13/2021) showed approximately a 4% difference in their nucleotide sequence compared to the VP6 of the reference strain (GenBank Accession Number MZ327693.1), which included a total of 45 and 47 nucleotide substitutions for RVB/Horse-wt/USA/KY1-6/2021 and RVB/Horse-wt/USA/KY1-13/2021, respectively. Among these, three and one nucleotide substitutions were located in the probe (ERVB-VP6-P) and reverse primer (ERVB-VP6-R) sequences, respectively (G_196_ → T_196_; C_202_ → T_202_; G_208_ → A_208_; G_217_ → A_217_; Figure 3). In spite of these differences, the ERVA/ERVB VP6 quadruplex assay was able to readily detect RVB/Horse-wt/USA/KY1-13/2021, while RVB/Horse-wt/USA/KY1-6/2021 yielded undetermined results. Since the assay was still able to amplify one of these samples despite the nucleotide substitutions within the probe and reverse primer sequences, a sample-specific PCR inhibitor was suspected in this case.

Finally, we compared the sensitivity for each of the targets in common between this ERVA/ERVB VP6 quadruplex assay and that of our previously described ERVA triplex assay. The NSP3 (pan-RVA) assay was the only ERVA-specific target which showed a slightly reduced sensitivity (91.8%, 7/85 positive samples that yielded either Ct values >35 [n = 2] or undetermined results [n = 5]) compared with the previously described ERVA triplex assay (*p*-value = 0.0083) in which sensitivity was 100% [58] (Table 5). For G3 and G14 targets, the sensitivities as determined with the ERVA/ERVB VP6 quadruplex assay were statistically equivalent to those reported for the ERVA triplex assay (*p*-values > 0.05) [58] (Table 5). The specificities in all cases were 100%.

## 4. Discussion

Group A rotaviruses continue to be a significant cause of diarrhea in children and animal species, including horses [1,2,3,4,5,7,8,66,67]. Based on the antigenic variations between circulating ERVA genotypes, their spatiotemporal distribution and their consequent impact on vaccine efficacy, surveillance and genotypification of circulating strains are necessary to inform on the need for updated vaccines for control and prevention. Most recently, outbreaks of diarrhea in foals associated with ERVB have been detected in Central Kentucky [10]. However, this rotavirus group has been only reported in one out of 37 fecal samples in a single study from Germany [48] and little is known about its distribution, prevalence and pathogenicity compared to ERVA. This new occurrence highlights the potential of this virus to emerge as a pathogen and, consequently, diagnostic and epidemiology tools are imperative to understand its biology, epidemiology, virulence, evolution, and ability to generate reassortants. Recently, singleplex TaqMan^®^ RT-qPCR assays for ERVB have been described [10] but these have not been thoroughly evaluated or incorporated into existing assays for equine rotavirus diagnostics and surveillance.

With the identification of ERVB in the US, we have modified our previously developed ERVA triplex assay [58] and incorporated an ERVB-specific target (VP6 or NSP5) to generate a quadruplex assay for the simultaneous differentiation between ERVA and ERVB, and genotyping of ERVA strains in a single reaction using the TaqMan^®^ chemistry. Thus, this study reveals the flexibility of this assay to rapidly adapt to the needs of the equine industry. The newly developed assays (ERVA/ERVB VP6 quadruplex and ERVA/ERVB NSP5 quadruplex) showed a 10-fold higher detection rate limit for the ERVA-specific targets compared to the previously developed ERVA triplex assay [58]; this difference could be associated with the modified probe design with optimized dyes and incorporation of a minor groove binder (MGB) in the design of the G3 VP7-specific probes. When compared to singleplex assays, however, the LOD_95%_ is approximately 10-fold higher on the quadruplex assays; the source of this is likely related to the differences in PCR efficiency for the ERVB targets between single and quadruplex formats. While the two assays developed and evaluated here (ERVA/ERVB VP6 and ERVA/ERVB NSP5) had comparable analytical performance, ERVA/ERVB VP6 was selected for clinical performance evaluation over the ERVA/ERVB NSP5 based on its overall higher efficiency among all the targets included in the assay. The overall sensitivity of the assay for all targets based on its clinical performance was 94% with a specificity of 100%. The sensitivity of the pan-RVA (NSP3) assay was 91.8%, slightly but significantly lower when compared to the previously developed triplex assay. Among the n = 7 misidentified samples, (a) n = 2 had Ct values of 38 and 39, respectively, with undetermined genotyping results; (b) n = 4 yielded an undetermined result but positive detection by the G3/G14 genotyping primer-probe set; (c) n = 1 yielded undetermined results for all targets. We have further tested the latter specimen using a spike-in internal control, which yielded a Ct of ~27, thus indicating that PCR inhibitors are unlikely to be the source of the failed amplification. Therefore, for the scenario presented under (a) and (c), compromised target integrity is a likely possibility that could have accounted for these results. The scenario presented under (b) could be associated with target competition and exhaustion of reagents during the reaction, which could impact the assay’s sensitivity [68]; these would still be considered positive following amplification of either G3 or G14. While these discordant samples have caused a slightly reduced sensitivity of the pan-RVA (NSP3) component of the assay, the sensitivity is still >90%. Although the NSP3 target showed this lower sensitivity in the ERVA/ERVB VP6 quadruplex assay compared to the triplex assay previously developed, the G3 and G14 VP7 targets had comparable sensitivity. Reduced sensitivity of multiplex RT-qPCR or qPCR assays compared to singleplex counterparts is not unusual and has been previously reported [69,70,71,72,73,74,75,76,77]. Based on previous studies, the reduction in sensitivity compared to singleplex assays is typically slight and could be due to differential amplification of one target over others (based on the amplification efficiencies), target abundance, reagent competition, and non-specific interactions between primer sets or a combination of these [70,71,72,74]. In our previous study, we demonstrated that, in case of low target concentration and high Ct values on the NSP3 assay with no amplification of either genotyping target G3 or G14, genotyping performance can be improved in those cases by performing them under singleplex. This only occurred in a small subset of samples analyzed (3 out of 177; 1.75%) [58]. It is important to note that, in the current study, the three G3 ERVA-positive samples that yielded negative results were the same samples that failed to be genotyped on our previously developed ERVA triplex assay [58]. Thus, low target nucleic acid in these fecal specimens beyond the limit of detection is suspected. Despite this, and with only one out of 193 samples included in the current study (0.52%) in which none of the assays’ targets amplified, the quadruplex assay developed here clearly offers a robust, fast, streamlined, and superior tool for surveillance and diagnosis of equine rotaviruses compared to other available tools such as conventional RT-PCR coupled with sequencing, antigen-based enzyme-linked immunosorbent assays (ELISA), or TEM.

Even though a small number of positive samples for ERVB (n = 15) could be included in this study, the ERVA/ERVB VP6 quadruplex assay was able to correctly detect ERVB in all except one fecal sample (RVB/Horse-wt/USA/KY1-6/2021). VP6 sequencing demonstrated that two of the samples including the one mentioned above showed roughly 4% nucleotide divergence from the reference strain and other ERVB-positive samples sequenced in this study, with a total of four nucleotide substitutions spanning the ERVB VP6 probe and reverse primer sequences used (n = 3 and n = 1, respectively). However, these differences are unlikely to be the source of the negative result as one of the samples was readily detected by the ERVA/ERVB VP6 quadruplex assay. Additionally, PCR inhibitors were ruled out on this sample as indicated above. Hence, low target abundance could have been responsible for failed amplification.

## 5. Conclusions

In conclusion, this newly developed quadruplex RT-qPCR assay (ERVA/ERVB VP6) demonstrates to be a robust, reliable, and rapidly adaptable tool for the diagnosis and surveillance of ERVA and ERVB in the field. Its flexibility allows for rapid expansion to include other emergent ERVA and ERVB types in the future.

## Figures and Tables

**Table 1:** Primers and probe combinations for the detection of rotavirus A (pan-rotavirus A, targeting the NSP3 gene), VP7 gene of ERVA G3 and G14 genotypes, VP6 gene of ERVB and NSP5 gene of ERVB. The fluorescent dyes and quenchers are in bold.

Name	Target	Nucleotide Position	Sequence (5′ to 3′)
NVP3-FDeg ^1^	RVA NSP3	963–982 ^a^	ACCATCTWCACRTRACCCTC
NVP3-R1 ^1^	RVA NSP3	1053–1034 ^a^	GGTCACATAACGCCCCTATA
NVP3-Probe ^1^	RVA NSP3	984–1026 ^a^	**JUN**-ATGAGCACAATAGTTAAAAGCTAACACTGTCAA-**QSY**
RVA-G3-756F	ERVA VP7 (G3) ^b^	756–777	GATGTTACCACGACCACTTGTA
RVA-G3-872R	ERVA VP7 (G3) ^b^	872–854	AGTTGGATCGGCCGTTATG
RVA-G3-779P	ERVA VP7 (G3) ^b^	779–823	**FAM**-TGGGACCACGAGAGAATGTAGCTGT-**MGB**
RVA-G14-ARG869F	ERVA VP7 (G14) ^c^	869–885	ATCCGACTACGGCTCCA
RVA-G14-ARG1011R	ERVA VP7 (G14) ^c^	1011–990	TGCAGCAGAATTTAATGATCGC
RVA-G14-ARG886P	ERVA VP7 (G14) ^c^	886–915	**VIC**-CAGATTGGACGAATGATGCGTATAAATTGG-**MGB**
ERVB-VP6-F	ERVB VP6	132–153 ^d^	CATCCAGAGTGAATGGGAAGAC
ERVB-VP6-R	ERVB VP6	230–210 ^d^	TTCTAACGGCCAGCGAAATTA
ERVB-VP6-P	ERVB VP6	187–209 ^d^	**LIZ**-CCCTTACACGATACACGCACCGA-**QSY**
ERVB-NSP5-F	ERVB NSP5	124–146 ^e^	GCCTTCTGATTCTACGTCAACTA
ERVB-NSP5-R	ERVB NSP5	238–215 ^e^	CTTGTTGTACGCTTCTTCGTATTC
ERVB-NSP5-P	ERVB NSP5	160–183 ^e^	**LIZ**-AACATCAAGTCGTAGCGACGCAGT-**QSY**

^1^ Primers and probe name and sequences derived from Freeman et al., 2008 [63]. ^a^ nucleotide position based on GenBank Accession number X81436. ^b^ nucleotide position based on GenBank Accession number KM454497.1. ^c^ nucleotide position based on GenBank Accession number KM454508.1. ^d^ nucleotide position based on GenBank Accession number MZ327693.1. ^e^ nucleotide position based on GenBank Accession number MZ327698.1. FAM, 6-carboxyfluorescein; JUN, JUN™ dye; LIZ, LIZ™ dye; MGB, minor groove binder; QSY, QSY™ quencher; VIC, VIC™ dye.

**Table 2:** Analytical performance of singleplex ERVA-specific, singleplex ERVB-specific, and quadruplex ERVA/ERVB-specific RT-qPCR assays for the detection and genotyping of equine rotavirus A and detection of equine rotavirus B (VP6 or NSP5).

	ERVA Singleplex			ERVB Singleplex		Quadruplex (ERVA/ERVB VP6)				Quadruplex (ERVA/ERVB NSP5)			
Parameter	G3	G14	NSP3	VP6	NSP5	G3	G14	NSP3	VP6	G3	G14	NSP3	NSP5
Slope	−3.3936	−3.3732	−3.2533	−3.4215	−3.4088	−3.1487	−3.3054	−3.3159	−3.496	−3.3288	−3.4355	−3.4354	−3.3723
Linearity (*R^2^*)	>0.99	>0.99	>0.99	>0.99	>0.99	>0.99	>0.99	>0.99	>0.99	>0.99	>0.99	>0.99	>0.99
Efficiency (%)	97	98	103	96	96.5	108	100	100	93.22	100	95	95	98
LOD_95%_ (copies/μL)	2.6	5.7	27	20	20	67	67	67	747	67	67	67	747
Detection rate limit (100%, copies/μL)	10	10	100	100	100	100	100	100	1000	100	100	100	1000
Ct cut-off	38	39	34	36	37	34	39	35	34	35	36	34	35

LOD_95%_, limit of detection 95%; Ct, cycle threshold. ERVA singleplex parameters have been previously analyzed and reported [58].

**Table 3:** Precision evaluation of the ERVA/ERVB VP6-specific and ERVA/ERVB NSP5-specific multiplex RT-qPCR assays. (a) Within-run and (b) between-run imprecision. Values represent the coefficient of variation in %.

**(a) Within-run**	**ERVA/ERVB VP6 Quadruplex Assay**				**ERVA/ERVB NSP5 Quadruplex Assay**			
**Concentration of target (IVT RNA copies/μL)**	**G3**	**G14**	**NSP3**	**VP6**	**G3**	**G14**	**NSP3**	**NSP5**
100,000	0.55%	0.43%	0.66%	0.33%	0.86%	0.91%	0.77%	0.51%
10,000	1.62%	1.05%	1.11%	0.53%	1.11%	0.85%	1.91%	0.97%
1000	0.89%	1.68%	1.87%	1.11%	0.51%	1.3%	1.99%	0.99%
**(b) Between-run**	**ERVA/ERVB VP6 Quadruplex Assay**				**ERVA/ERVB NSP5 Quadruplex Assay**			
**Concentration of target (IVT RNA copies/μL)**	**G3**	**G14**	**NSP3**	**VP6**	**G3**	**G14**	**NSP3**	**NSP5**
100,000	1.2%	1.1%	0.30%	0.32%	1.2%	1.1%	0.66%	0.30%
10,000	0.95%	1.23%	1.35%	0.45%	1.05%	1.14%	1.77%	0.80%
1000	1.07%	2.31%	2.17%	0.90%	0.46%	0.93%	1.03%	0.59%

**Table 4:** Evaluation of the clinical performance of the ERVA/ERVB VP6 quadruplex RT-qPCR assay for the detection and genotyping of ERVA/ERVB in fecal samples compared to ERVA VP7-specific RT-PCR or ERVB VP6-specific RT-PCR and sequencing (gold standard). (a) ERVA NSP3, (b) ERVA G3 VP7, (c) ERVA G14 VP7, and (d) ERVB VP6. Sensitivity, specificity, and agreement (with weighted kappa index, alpha’s standard error [ASE], and *p*-value) are indicated below.

**(a)**		**ERVA VP7-Specific RT-PCR**		
		**Positive**	**Negative**	**Total**
**NSP3-specific RT-qPCR**	**Positive**	78	0	78
	**Negative**	7	108	115
	**Total**	85	108	193
**(b)**		**ERVA Genotype G3 ^1^**		
		**Positive**	**Negative**	**Total**
**G3-specific RT-qPCR**	**Positive**	38	0	38
	**Negative**	3	152	155
	**Total**	41	152	193
**(c)**		**ERVA Genotype G14 ^1^**		
		**Positive**	**Negative**	**Total**
**G14-specific RT-qPCR**	**Positive**	44	0	44
	**Negative**	1	148	149
	**Total**	45	148	193
**(d)**		**ERVB VP6-Specific RT-PCR**		
		**Positive**	**Negative**	**Total**
**VP6-specific RT-qPCR**	**Positive**	14	0	14
	**Negative**	1	178	179
	**Total**	15	178	193

(a) Sensitivity: 91.8%; Specificity: 100%; Agreement: 96.4% (weighted kappa = 0.926; ASE = 0.027; *p*-value < 0.0001). (b) ^1^ Genotype determined by Sanger sequencing; Sensitivity: 92.7%; Specificity: 100%; Agreement: 98.5% (weighted kappa = 0.926; ASE = 0.027; *p*-value < 0.0001). (c) ^1^ Genotype determined by Sanger sequencing; Sensitivity: 97.8%; Specificity: 100%; Agreement: 99.5% (weighted kappa = 0.985; ASE = 0.015; *p*-value < 0.0001). (d) Sensitivity: 93.3%; Specificity: 100%; Agreement: 99.5% (weighted kappa = 0.963; ASE = 0.037; *p*-value < 0.0001).

**Table 5:** Comparison of the clinical sensitivity for the targets in common between the previously reported ERVA triplex RT-qPCR assay and the ERVA/ERVB VP6 quadruplex RT-qPCR assay.

Target	ERVA Triplex Sensitivity	ERVA/ERVB VP6 Quadruplex Sensitivity	*p*-Value *
NSP3	100%	91.8%	0.0083
G3	92.7%	92.7%	1
G14	100%	97.8%	0.3

* McNemar’s test; statistical significance set at *p*-value < 0.05.

## Data Availability

The nucleotide sequences derived from the fecal samples utilized in this study were deposited in GenBank under accession numbers OP314521-OP314535.

## Supplementary Materials

The following supporting information can be downloaded at: https://www.mdpi.com/article/10.3390/v15081626/s1, Table S1: Primers used for RT-PCR amplification and sequencing of VP7 (genome segment 9) of ERVA and VP6 (genome segment 6) of ERVB. Figure S1: Negative staining of equine rotavirus B (ERVB) particles in fecal specimens. Clustered (A) or individualized (B) viral particles were identified in fecal specimens. ERVB particle size ranged from 48.2 to 62.4 nm and showed a typical “wheel” shape with spike-like projections from the outer capsid as shown in (A) and (B). Transmission electron microscopy, 40,000× magnification. Bar = 50 μm.
