# Supplementary material for: Quadruplex Real-Time TaqMan® RT-qPCR Assay for Differentiation of Equine Group A and B Rotaviruses and Identification of Group A G3 and G14 Genotypes
Source: Viruses. 2023 Jul 26;15(8):1626. doi: 10.3390/v15081626 (PMC10459720; doi:10.3390/v15081626)
Supplement: Supplementary file 1 [file viruses-15-01626-s001.zip › Supplementary Table S1.pdf]

Supplementary Table S1. Primers used for RT-PCR amplification and sequencing of VP7 (genome segment 9) of ERVA and VP6 (genome segment 6) of ERVB

Primer name	Target	Nucleotide Position	Sequence (5′ to 3′)	Application
RVAVP7-Gra-5	ERVA VP7	1-20[a]	GGCTTTAAAAGCGAGAATTT	RT-PCR and sequencing
RVAVP7-Gra-3	ERVA VP7	1,062-1,044[a]	GGTCACATCATACAACTCT	RT-PCR and sequencing
RVAVP7-389-R	ERVA VP7	389-370[a]	CCAGTAGGCCATCCTTTAGT	Sequencing
RVAVP7-635-F	ERVA VP7	635-659[a]	GTCCACTTAATACACAAACTCTAGG	Sequencing
RVAVP7-241-R	ERVA VP7	245-220[a]	GCAGTRTCCATTGAACCAGTAATTG	Sequencing
RVAVP7-852-F	ERVA VP7	856-879[a]	GAYATAACGGCTGATCCAACTACG	Sequencing
RVAVP7-881-F	ERVA VP7	885-906[a]	CTCCACAGATTGGACGAATGA	Sequencing
RVBVP6-29-F	ERVB VP6	29-48[b]	GTGAACGCTTGCGTCAGATT	RT-PCR and sequencing
RVBVP6-1201-R	ERVB VP6	1,201-1,182[b]	CGGGCTCAGGGAGAATCAGA	RT-PCR and sequencing
RVBVP6-744-F	ERVB VP6	744-763[b]	CCGCAGAGATGACAGCAGAT	Sequencing
RVBVP6-455-R	ERVB VP6	455-436[b]	CTGGTTGGATAAGCGCGGTA	Sequencing

[a]nucleotide position based on GenBank Accession number KM454508.1
[b]nucleotide position based on GenBank Accession number MZ327693.1
