# Supplementary figures and images for: Quadruplex Real-Time TaqMan® RT-qPCR Assay for Differentiation of Equine Group A and B Rotaviruses and Identification of Group A G3 and G14 Genotypes
Source: Viruses. 2023 Jul 26;15(8):1626. doi: 10.3390/v15081626 (PMC10459720; doi:10.3390/v15081626)

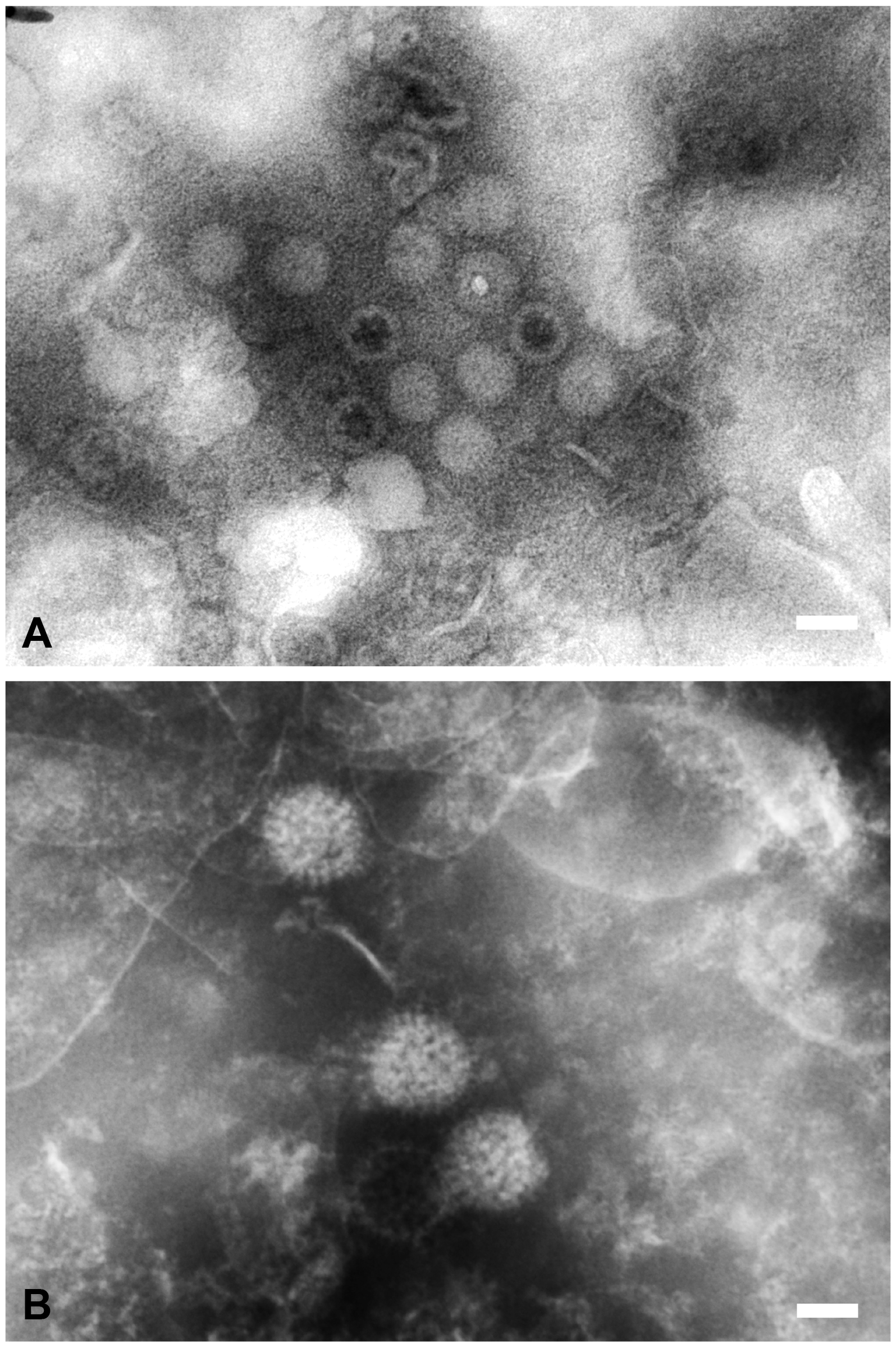

Supplement: Supplementary file 1 [file viruses-15-01626-s001.zip › Supplementary Figure S1.tif]
